# Rapid Activation of Transforming Growth Factor β–Activated Kinase 1 in Chondrocytes by Phosphorylation and K^63^‐Linked Polyubiquitination Upon Injury to Animal Articular Cartilage

**DOI:** 10.1002/art.39965

**Published:** 2017-02-27

**Authors:** Heba M. Ismail, Athanasios Didangelos, Tonia L. Vincent, Jeremy Saklatvala

**Affiliations:** ^1^ University of OxfordOxfordUK; ^2^ King's College LondonLondonUK

## Abstract

**Objective:**

Mechanical injury to cartilage predisposes to osteoarthritis (OA). Wounding of the articular cartilage surface causes rapid activation of MAP kinases and NF‐κB, mimicking the response to inflammatory cytokines. This study was undertaken to identify the upstream signaling mechanisms involved.

**Methods:**

Cartilage was injured by dissecting it from the articular surface of porcine metacarpophalangeal (MCP) joints or by avulsing murine proximal femoral epiphyses. Protein phosphorylation was assayed by Western blotting of cartilage lysates. Immunolocalization of phosphorylated activating transcription factor 2 (ATF‐2) and NF‐κB/p65 was detected by confocal microscopy. Messenger RNA (mRNA) was measured by quantitative reverse transcriptase–polymerase chain reaction (qRT‐PCR). Receptor associated protein 80 (RAP‐80) ubiquitin interacting motif agarose was used in a pull‐down assay to obtain K^63^‐polyubiquitinated proteins. Ubiquitin linkages on immunoprecipitated transforming growth factor β–activated kinase 1 (TAK‐1) were analyzed with deubiquitinases.

**Results:**

Sharp injury to porcine cartilage caused rapid activation of JNK and NF‐κB pathways and the upstream kinases MKK‐4, IKK, and TAK‐1. Pharmacologic inhibition of TAK‐1 in porcine cartilage abolished JNK and NF‐κB activation and reduced the injury‐dependent inflammatory gene response. High molecular weight species of phosphorylated TAK‐1 were induced by injury, indicating its ubiquitination. An overall increase in K^63^‐linked polyubiquitination was detected upon injury, and TAK‐1 was specifically linked to K^63^‐ but not K^48^‐polyubiquitin chains. In mice, avulsion of wild‐type femoral epiphyses caused similar intracellular signaling that was reduced in cartilage‐specific TAK‐1–null mice. Epiphyseal cartilage of MyD88‐null and TRAF‐6–null mice responded to injury, suggesting the involvement of a ubiquitin E3 ligase other than TRAF‐6.

**Conclusion:**

Activation of TAK‐1 by phosphorylation and K^63^‐linked polyubiquitination by injury indicates its role in driving cell activation. Further studies are needed to identify the upstream ubiquitination mechanisms, including the E3 ligase involved.

Traumatic injury to vascular tissues causes inflammation and wound healing, but how tissues sense mechanical injury is not understood. A prevailing view is that sterile inflammation is promoted by damage causing release of intracellular materials such as ATP, S100A proteins, heat‐shock proteins, and high mobility group box chromosomal protein 1, the latter acting via Toll‐like receptors (TLRs) to induce an inflammatory response 1, 2, 3, 4, 5, 6. In this view, normal constituents released from damaged or stressed cells act as mediators analogously to microbial pathogen–associated molecular patterns and have been termed damage‐associated molecular patterns (DAMPs). The cellular players have not been characterized, but it would follow that macrophages would respond to DAMPs and orchestrate inflammation.

We have studied the effects of mechanical injury on articular cartilage because such injury predisposes to osteoarthritis (OA). Simply cutting the articular cartilage surface from porcine metacarpophalangeal (MCP) joints has been shown to cause rapid intracellular signaling with activation of NF‐κB, MAP kinases 7, 8, protein kinase B, and Src, followed by induction of inflammatory response genes 9. The response resembled those elicited by bacterial lipopolysaccharide (LPS) or inflammatory cytokines such as interleukin‐1 (IL‐1) and tumor necrosis factor (TNF), but we found no soluble mediator arising from the injured tissue to explain it. An interesting feature was that cutting the cartilage again in culture, having allowed the first response to die down, did not induce the full range of inflammatory signaling, but induced only activation of ERK, due to release of pericellular fibroblast growth factor 7, 10, 11. Thus, the tissue in vivo had an alarm that was activated by injury but was not reset in culture. Fresh tissue is therefore needed for experiments.

Synovium is a vascular connective tissue that has a variety of cell types, but shows the same pattern of intracellular signaling upon cutting injury 9, suggesting that the response is generic. After working with porcine tissue, we studied mice to see if their response was the same. It is not possible to make discrete scalpel injuries to murine articular cartilage in quantities sufficient for analysis. Instead, we avulsed the proximal femoral epiphysis from 5‐week‐old animals. This injury shears through the cartilaginous growth plate. The same pattern of activation of MAP kinases, NF‐κB, and inflammatory response genes as was seen in porcine tissue occurred 12.

The role of inflammation in OA is uncertain, but since mechanical injury predisposes to OA and directly activates inflammatory signaling, it seemed important to understand the molecular mechanism of the response. Further evidence of the relevance of inflammatory signaling is that the MAPK/JNK pathway controls aggrecan degradation by chondrocytes 13, and in OA induced by destabilizing the knee, JNK‐2–null mice showed reduced aggrecan degradation and slower disease progression 14.

The inflammatory triggers whose mechanisms are best understood are LPS, IL‐1, and TNF. These signal via their receptors through transforming growth factor β–activated kinase 1 (TAK‐1) 15, 16. TAK‐1 is bound to TAB‐1 17, 18, 19, which binds to K^63^‐linked polyubiquitin chains whose formation is triggered by activation of the receptors. Binding of the TAK‐1/TAB‐1 complex to K^63^‐polyubiquitin chains facilitates TAK‐1 autophosphorylation and activation 20, 21. The γ subunit of the IKK complex also binds to K^63^‐polyubiquitin. TAK‐1 then activates the IKKs and MAP kinase kinases.

In LPS and IL‐1 signaling, the K^63^‐polyubiquitination is carried out by TNF receptor–associated factor 6 (TRAF‐6), a ubiquitin E3 ligase, working in conjunction with the E2 complex Ubc13/Uev1A, and E1 22, 23. TLR‐4 (the LPS receptor) and IL‐1 receptor (IL‐1R) are homologous and when liganded interact with the adaptor protein myeloid differentiation factor 88 (MyD88). IL‐1R–associated kinases bind to these receptor complexes and activate TRAF‐6. The K^63^‐polyubiquitin chains formed may be free or attached to proteins, including TRAF‐6 itself, IRAK, and TAK‐1 24. TNF receptor type I also signals via TAK‐1 but uses TRAF‐2 and TRAF‐5 as E3 ligases 25. K^63^‐polyubiquitination causes proximity‐induced signaling 26 in contrast to K^48^‐polyubiquitination, which targets proteins for degradation 27.

In this study, we have shown that mechanical injury to cartilage activates NF‐κB and MAP kinases via TAK‐1, that there is formation of K^63^‐polyubiquitin chains, and TAK‐1 is a target. The polyubiquitination pattern after injury is distinct from that caused by either IL‐1 or TNF, and TRAF‐6 is not involved. Thus, chondrocytes respond directly to local mechanical injury of cartilage with signaling and gene expression characteristic of inflammation, which is driven by an unidentified ubiquitin E3 ligase and could cause cartilage destruction in OA.

## MATERIALS AND METHODS

### Reagents and antibodies

Anti–TAK‐1 (catalog no. D94D7), anti–MKK‐4 (catalog no. 9152S), anti–phospho–MKK‐4T261 (catalog no. 9151S), anti‐IκB (catalog no. 4814S), anti–phospho‐IκB, Ser32/36 (catalog no. 9246), anti–phospho–p38‐T180/Y182 (catalog no. 9211S), anti–phospho–TAK‐1‐T184/187 (catalog no. 45085), anti–phospho–IKKα/β‐Ser176/180 (catalog no. 16A6), and anti–phospho–activating transcription factor 2 (ATF‐2)–Thr71 (catalog no. 9221) were from Cell Signaling Technology. Anti–phospho–JNK‐pTpY183/185 (catalog no. 44682G) was obtained from Invitrogen. Anti–T‐ERK (catalog no. sc‐94), anti–NF‐κB–p65 (catalog no. Sc‐372), anti–TAK‐1 (catalog no. sc‐7162), and anti–phospho–TAK‐1‐S192 (catalog no. sc‐130219) were from Santa Cruz Biotechnology. Anti‐ubiquitin–Lys‐63–specific antibody (catalog no. 05‐1308) was from Millipore. Myelin basic protein (MBP; catalog no. M1891) and anti–phospho‐ERK (catalog no. M9692) were from Sigma. A ubiquitin chain restriction enzyme analysis (UbiCREST) kit (catalog no. K‐400) was obtained from R&D Systems, and γ^32^P‐ATP was obtained from PerkinElmer. TAK‐1 inhibitor 5z‐7‐oxozeanol (catalog no. 3604) was from Tocris.

### Cartilage injury and culture

#### Porcine cartilage

Articular cartilage was obtained from the MCP joints of the forefeet of freshly slaughtered 3–6‐month‐old pigs purchased from a local abattoir. Forefeet were first decontaminated in 2% Virkon, then equilibrated at 37°C. MCP joints were opened and injury to cartilage was induced by rapidly dissecting it from the articular surface and cutting it into small pieces (1–2 mm^3^) as previously described 9. Cartilage was either snap‐frozen (at time point 0) or cultured for various amounts of time in serum‐free medium. In some experiments, joints were first injected with 5 μ*M* TAK‐1 inhibitor (2 ml/joint) or DMSO in Dulbecco's modified Eagle's medium and then equilibrated at 37°C for 1 hour before dissection.

#### Murine femoral epiphysis

Mice (5–6 weeks old) were culled by cervical dislocation. Injury to the cartilaginous epiphysis was induced after section of the acetabulofemoral ligament by avulsing the epiphysis with forceps as previously described 12. The epiphyseal cartilage was either snap‐frozen or cultured for various amounts of time in serum‐free medium.

Porcine and murine cartilage samples were then lysed in 1× radioimmunoprecipitation assay (RIPA) buffer for Western blotting or used for RNA extraction and inflammatory gene expression analysis as previously described 9.

### TAK‐1 immunoprecipitation

Protein G magnetic beads (Invitrogen) were washed with 1× RIPA buffer. TAK‐1 was then immunoprecipitated from cartilage lysates using TAK‐1–specific antibody (1:250) overnight at 4°C in the presence of 50 μl/ml protein G magnetic beads, with rotation. Beads were then centrifuged and washed with 1× RIPA buffer, and immunoprecipitates were analyzed by Western blotting or kinase activity was assessed by UbiCREST assay.

### TAK‐1 kinase assay

TAK‐1 was immunoprecipitated from cartilage lysates as described above (∼0.5 gm cartilage per time point). Beads were washed 3 times in 1× RIPA buffer and washed once in 50 m*M* Tris HCl (pH 7.5). Beads were then resuspended in 25 μl of freshly prepared kinase buffer (50 m*M* Tris HCl [pH 7.5], 100 μ*M* EGTA, 100 μg/ml bovine serum albumin, 10 m*M* MgCl_2_, 100 μ*M* Na_3_VO_4_, 2 m*M* EDTA, 1 μ*M* microcystin, and10 μ*M* ATP) supplemented with 2 µg of MBP as substrate and 5 μCi γ^32^P‐ATP. Reactions were carried out at 30°C for 30 minutes and then terminated by adding 15 μl of 5× sodium dodecyl sulfate–polyacrylamide gel electrophoresis (SDS‐PAGE) sample buffer and boiling for 5 minutes. Samples were then analyzed by SDS‐PAGE, and the amount of ^32^P incorporated into MBP was assessed by autoradiography and quantified by phosphorimaging (Fujifilm FLA‐5100 phosphorimager).

### Pull‐down assay for polyubiquitinated proteins using receptor associated protein 80 (RAP‐80) ubiquitin interacting motifs (UIMs)

UIMs in RAP‐80 preferentially bind K^63^‐linked ubiquitin. RAP‐80–UIM resin was equilibrated by washing with 1× RIPA buffer. Fifty microliters of washed resin was incubated with 1 ml of cartilage lysates overnight at 4°C with rotation. RAP‐80 resin was then centrifuged at 2,800 revolutions per minute for 3 minutes at 4°C and washed 3 times with 1× RIPA buffer. Beads were resuspended in 1× sample buffer, and the bound proteins were analyzed by Western blotting using anti–K^63^ ubiquitin‐specific antibody.

### Ubiquitin chain restriction enzyme analysis

A UbiCREST kit was used to identify the type of ubiquitin linkages bound to TAK‐1, according to the recommendations of the manufacturer. Briefly, TAK‐1 was immunoprecipitated from cartilage lysates as described above. Cartilage lysates from 3 joints were used for each set of assays. Immunocomplexes were then washed 3 times with RIPA buffer and 1 time with deubiquitinase dilution buffer. Beads were then divided into 9 equal parts. In 50 μl deubiquitinase buffer aliquots, 5 μl of a deubiquitinase was added to each tube and incubated for 30 minutes at 37°C. Tubes were then centrifuged, and supernatants were used for primary analysis by SDS‐PAGE and silver staining of released ubiquitin chains, while the beads were analyzed for the efficiency of deubiquitinase enzyme cleavages by Western blotting. The deubi‐quitinase enzymes have the following linkage specificity: OTUD3 cleaves K^6^‐ and K^11^‐linked polyubiquitin; CEZANNE cleaves K^11^‐linked polyubiquitin only; His6‐TARBID cleaves K^29^‐, K^33^‐, and K^63^‐linked polyubiquitin; otubain 1 (OTUB‐1) cleaves K^48^‐linked polyubiquitin only; GST‐AMSH cleaves K^63^‐linked polyubiquitin only; YOD1 cleaves K^6^‐, K^11^‐, K^27^‐, K^29^‐, and K^33^‐linked polyubiquitin; OTULIN cleaves linear polyubiquitin only; and USP2 cleaves most ubiquitin linkages (used as a positive control).

### NF‐κB and phospho–ATF‐2 immunofluoresence staining

Full‐thickness porcine cartilage explants were snap‐frozen in liquid nitrogen immediately after completion of the experiment (immediately after injury for time point 0 and immediately after incubation for the 30‐minute time point). Explants were then cryosectioned using a cryostat (CM‐1900; Leicia Microsystems) operated at −20°C. Cartilage sections were air dried at room temperature for 1 hour, fixed with 4% paraformaldehyde in 1× phosphate buffered serum (PBS) for 20 minutes, and then washed briefly with 1× PBS. Cells were permeabilized with 0.2% Triton X‐100 in 1× PBS for 20 minutes. Sections were blocked in 10% goat serum for 1 hour and then incubated with primary antibodies (1:100) overnight at 4°C. Following incubation, sections were washed with 1× PBS, incubated with Alexa Fluor 488–conjugated secondary antibodies for 2 hours and 2 μg/ml propidium iodide, and then washed and mounted in 50% glycerol in 1× PBS. Signals were visualized by Ultraview confocal microscopy (Nikon Eclipse TE2000U).

### Animals

C57BL/6 (wild‐type) mice were purchased from Charles River. MyD88‐null and TAK‐1 (MAP3K7) loxP‐flanked (floxed) mice were originally acquired from The Jackson Laboratory. The CreER aggrecan promoter line 28 was generated at the Kennedy Institute by George Bou‐Gharios. Animal experiments were performed after ethics and statutory approval was obtained in accordance with local policy. Mice were maintained at 21°C in standard, individually ventilated cages holding 4–6 mice per cage.

TRAF‐6–null animals were generated at the Institute of Medical Science, University of Tokyo, and distributed from RIKEN BioResource Center. In TRAF‐6–knockout mice, a 2.5‐kb genomic fragment was replaced with a neomycin selection cassette. Heterozygous animals were used for breeding. Homozygous TRAF‐6–mutant mice have been reported to exhibit severe osteopetrosis and abnormal immune cell development and function, and die within ∼2 weeks after birth 29. In our experiments, homozygous offspring were very sick with breathing difficulties. In some instances there were no homozygous offspring.

TAK1^fl/fl^; AggCreER^T2^ cartilage‐specific–knockout mice were generated by crossing floxed TAK‐1 mice (TAK1^fl/fl^, B6;129S7‐Map3k7tm1Mds/J) with AggCreER^T2^ mice. All mice were on a C57BL/6J background, and their genotype was determined by polymerase chain reaction (PCR). Mutation of TAK‐1 and incorporation of aggrecan Cre was confirmed by PCR using the following primer sets: for TAK‐1, forward 5′‐AGCTCGCTGGTAGTGGTGTT‐3′ and reverse 5′‐GCCCCACTGTGAATCTGAAA‐3′; for AggCre, forward 5′‐GCATTACCGGTCGATGCAACGAGTGATGAG and reverse 5′‐GAGTGAACGAACCTGGTCGAAATCAGTGCG.

All mice (floxed, controls, and crossed lines) were injected intraperitoneally with tamoxifen (50 mg/kg) for 3 consecutive days at age 4–5 weeks. Two weeks after the last injection, mice were used in injury experiments. Loss of TAK‐1 was confirmed by Western blotting.

## RESULTS

### Rapid activation of the NF‐κB and JNK pathways and their upstream regulators in response to cartilage injury

**Figure 1:**
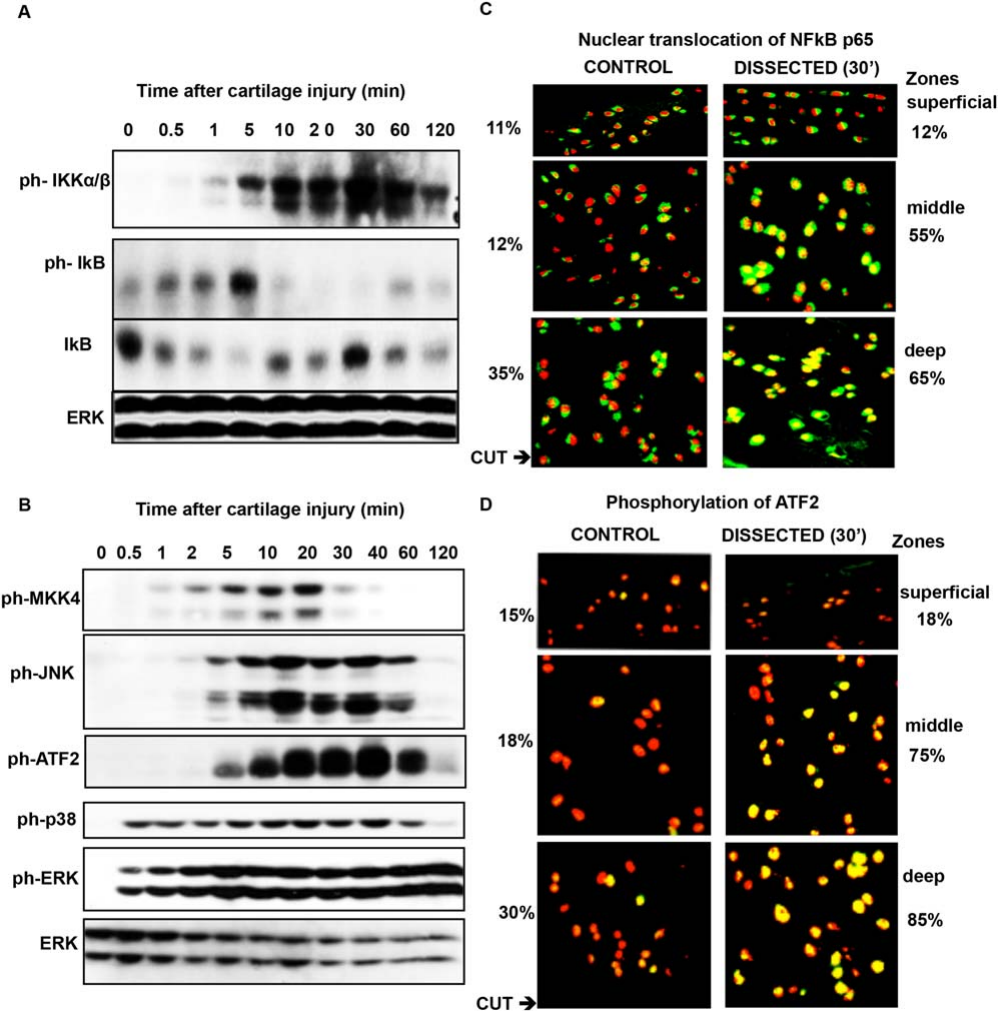
Activation of the NF‐κB and JNK pathways and their upstream regulators in injured porcine cartilage. Porcine metacarpophalangeal joints were equilibrated at 37°C for 1 hour. Articular cartilage was dissected and either snap‐frozen or kept in serum‐free medium for the indicated times. **A** and **B,** Cartilage was washed with 1× phosphate buffered saline and lysed in radioimmunoprecipitation assay buffer. Lysates were analyzed by Western blotting for phospho‐IKK (ph‐IKK), phospho‐IκB, and IκB (**A**) and for phospho‐MKK‐4, phospho‐JNK, phospho–activating transcription factor (phsopho–ATF‐2), phospho‐p38, and phospho‐ERK (**B**). ERK was blotted to check that the lanes were equally loaded. Results are representative of several replicate experiments. **C** and **D,** After incubation, cartilage was embedded in OCT, and cryosections were stained with antibodies against the p65 subunit of NF‐κB (**C**) or phospho‐ATF‐2 (**D**). The localization of the 2 proteins in different cartilage zones was determined using confocal microscopy. In each cryosection, a total of 500 cells were counted (100 cells in the superficial and deep zones and 300 in the middle zone). Percentages are the proportion of activated cells in the different zones. Representative images are shown. Original magnification × 60.

Dissection of porcine MCP joint cartilage from the articular surface induced rapid activation of the NF‐κB pathway. Phosphorylation of IKK was seen within 30 seconds and was maximal after 30 minutes. This was associated with degradation of IκB. IκB was resynthesized between 10 and 30 minutes, and the newly synthesized protein was phosphorylated and degraded (Figure 1A). Injury also rapidly activated the JNK pathway (Figure 1B). MKK‐4, a JNK activator, was phosphorylated by 1 minute, JNK itself by 2 minutes, and its substrates ATF‐2 (Figure 1B) and c‐Jun (results not shown) by 5 minutes. In addition, the 2 other MAP kinases, p38 and ERK, were phosphorylated within 30 seconds of injury (Figure 1B).

To determine whether activation of NF‐κB and JNK is propagated through the tissue, the extent of translocation of the p65 subunit of NF‐κB from cytoplasm to nucleus (Figure 1C), and the presence of phosphorylated ATF‐2 (Figure 1D), were analyzed by confocal microscopy of the deep, middle, and superficial zones of the cartilage ex vivo at time point 0 and 30 minutes after dissection and culture. The cut was made through the deep zone, so chondrocytes at the cut surface lie along the bottom of the deep zone panels shown in Figures 1C and D. NF‐κB p65 appears green in the cytoplasm and yellow in nuclei. Before injury, the majority of p65 was cytoplasmic (Figure 1C). The percentage of cells in which more than half of the nuclear area was yellow (an arbitrary measure of NF‐κB activation) is also shown in Figures 1C and D. After injury, increased nuclear translocation of NF‐κB p65 was seen throughout the deep and middle zones (Figure 1C), with the percentage of positive cells increasing 2 and 4 times, respectively. Phosphorylation of ATF‐2 (shown in yellow) was seen in nuclei and was much more pronounced in the deep and middle zones following injury, with the percentage of positive cells increasing by 2.5 and 4 times, respectively (Figure 1D).

### TAK‐1 is activated upon cartilage injury and inhibition of TAK‐1 abolishes injury‐dependent gene expression

**Figure 2:**
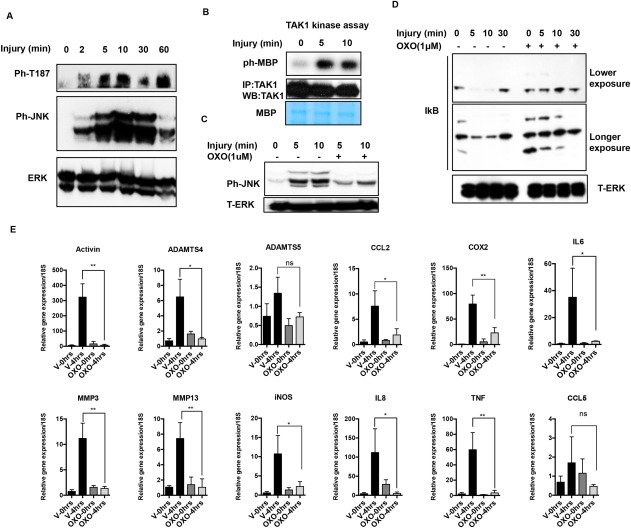
Cartilage injury leads to activation of transforming growth factor β–activated kinase 1 (TAK‐1), and inhibition of TAK‐1 abolishes injury‐dependent inflammatory gene expression. **A,** Porcine metacarpophalangeal (MCP) joints were equilibrated at 37°C for 1 hour. Articular cartilage was dissected and either snap‐frozen or kept in serum‐free medium for the indicated times. Cartilage was then washed with 1× phosphate buffered saline and lysed in radioimmunoprecipitation assay buffer. Lysates were analyzed by Western blotting (WB) for phospho–TAK‐1 (ph‐T187), phospho‐JNK, and ERK. **B,** A TAK‐1 activity assay was carried out as described in Materials and Methods to confirm TAK‐1 activation. IP = immunoprecipitation; ph‐MBP = phospho–myelin basic protein. **C** and **D,** Porcine MCP joints were injected with 5 μ*M* TAK‐1 inhibitor 5z‐7‐oxozeanol (OXO) or DMSO (vehicle [V]), as described in Materials and Methods, and kept at 37°C for 1 hour. Joints were then opened and cartilage was dissected and snap‐frozen (time point 0 minutes) or cultured for the indicated times with or without TAK‐1 inhibitor. Cartilage lysates were analyzed for phospho‐JNK and total ERK (T‐ERK) (**C**) and for IκB and total ERK (**D**). **E,** Cartilage was snap‐frozen (time point 0 hours) or kept at 37°C for 4 hours with or without 5 μ*M* TAK‐1 inhibitor (OXO). RNA was extracted from the tissue and used in quantitative reverse transcriptase–polymerase chain reaction studies of a panel of typical inflammatory response genes. Bars show the mean ± SEM of 3 independent experiments. ∗ = *P* < 0.05; ∗∗ = *P* < 0.01, by unpaired *t*‐test. NS = not significant; COX‐2 = cyclooxygenase 2; IL‐6 = interleukin‐6; MMP‐3 = matrix metalloproteinase 3; iNOS = inducible nitric oxide synthase; TNF = tumor necrosis factor. Color figure can be viewed in the online issue, which is available at http://onlinelibrary.wiley.com/journal/doi/10.1002/art.39965/abstract.

TAK‐1 activates JNK and NF‐κB in response to inflammatory stimuli. To investigate the role of TAK‐1 in injury, we analyzed its phosphorylation at T187 in the activation loop in lysates of cartilage made after increasing times following dissection. Western blotting showed that TAK‐1 was phosphorylated by 2 minutes after injury (Figure 2A). TAK‐1 activation was confirmed by radiolabeled kinase activity assay. For that assay, TAK‐1 was immunoprecipitated from lysates and assayed on MBP substrate. Incorporation of ^32^P from radiolabeled ATP was measured by autoradiography. Phosphorylation of MBP was induced by TAK‐1 from lysates made 5 and 10 minutes after injury (Figure 2B), confirming activation.

Next, we used a TAK‐1 inhibitor, 5z‐7‐oxozeanol 30, to see if inhibiting TAK‐1 affected injury‐dependent activation of JNK and NF‐κB and downstream gene induction. Porcine MCP joints were injected with TAK‐1 inhibitor or vehicle and kept at 37°C for 1 hour. The joints were then opened and cartilage was dissected and immediately snap‐frozen (time point 0), or kept in medium for various amounts of time, with or without 5z‐7‐oxozeanol. The TAK‐1 inhibitor abolished JNK phosphorylation (Figure 2C) and IκB degradation (Figure 2D) following injury. It also reduced induction of mRNAs for injury‐inducible genes (Figure 2E).

### Detection of higher molecular weight species of phosphorylated TAK‐1 in injured cartilage

**Figure 3:**
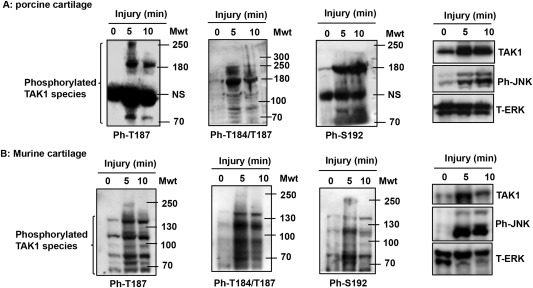
Higher molecular weight (Mwt) species of phosphorylated transforming growth factor β–activated kinase 1 (TAK‐1) in injured porcine and murine cartilage. **A,** Porcine metacarpophalangeal joints were processed as described in Figure 1. Cartilage was then dissected and either snap‐frozen (time point 0 minutes) or cultured for 5 or 10 minutes. Cartilage lysates were analyzed by Western blotting for TAK‐1 phosphorylated at T187 (ph‐T187), T184/T187, and S192, phospho‐JNK, and total ERK (T‐ERK; loading control). **B,** Murine proximal femoral epiphyses (2 per time point) were subjected to avulsion injury, and were then either snap‐frozen (time point 0 minutes) or cultured for 5 or 10 minutes in serum‐free medium. Epiphyses were analyzed by Western blotting for phospho–TAK‐1, phospho‐JNK, and total ERK (loading control) as described in **A**. Representative results from 4 independent experiments are shown. NS = nonspecific.

On Western blotting of lysates for phosphorylation of TAK‐1 T187, higher molecular weight phosphorylated species of ∼180 kd and 250 kd were seen in addition to the ∼80 kd form (Figure 3A). An antibody recognizing both T187 and T184 phosphorylation showed a more complex pattern suggestive of laddering, with the ∼180 kd species being most pronounced (Figure 3A). Phosphorylation of TAK‐1 at S192, which is also needed for maximal activation, was examined. The ∼80 kd band was induced by injury, but the ∼180 kd form was the major species, while the largest species was not clearly discerned (Figure 3A). These complex patterns suggested additional posttranslational modifications of TAK‐1, such as ubiquitination.

There was some increase in TAK‐1 protein levels (Figure 3A), possibly due to increased stability of TAK‐1 protein following injury.

### Overall increase in K^63^‐linked polyubiquitination in articular cartilage upon injury

**Figure 4:**
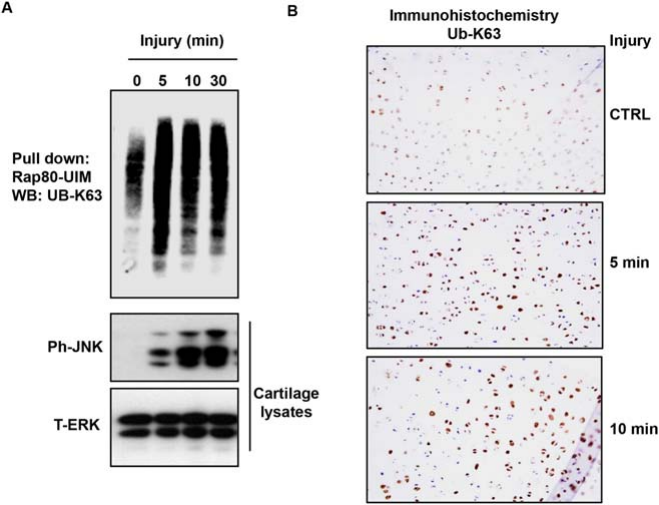
Increased K^63^‐linked polyubiquitin in injured porcine articular cartilage. Porcine metacarpophalangeal (MCP) joints were equilibrated at 37°C for 1 hour. **A,** Cartilage was dissected and either snap‐frozen (time point 0 minutes) or cultured for the indicated times. K^63^‐linked ubiquitin (UB‐K^63^) proteins were obtained from cartilage lysates using a pull‐down assay with receptor associated protein 80 (RAP‐80) ubiquitin interacting motifs (UIMs) as described in Materials and Methods, and proteins bound to the beads were analyzed by Western blotting (WB) with an anti–K‐63–linked ubiquitin antibody. Lysates were analyzed by Western blotting for phospho‐JNK (ph‐JNK) and total ERK (T‐ERK). Representative results from 3 independent experiments are shown. **B,** Porcine MCP cartilage sections (5–7 μm) were deparaffinized, rehydrated, and treated for 10 minutes with proteinase K for antigen retrieval. Endogenous peroxidase activity was blocked with 3% H_2_O_2_ for 10 minutes, followed by blocking with avidin–biotin for another 10 minutes. Sections were then blocked for 2 hours at room temperature with 10% goat serum in phosphate buffered saline, and incubated overnight at 4°C with anti‐K^63^ ubiquitin antibody (1:50 dilution). Sections were washed and then incubated for 2 hours in the dark with secondary antibodies conjugated with streptavidin. Antibody binding was visualized using a Vectastain kit and DAB Peroxidase Substrate Kit (Vector). Slides were then counterstained with hematoxylin and mounted with DPX. Original magnification × 10. Color figure can be viewed in the online issue, which is available at http://onlinelibrary.wiley.com/journal/doi/10.1002/art.39965/abstract.

To investigate the involvement of ubiquitination, lysates were made from injured porcine MCP cartilage, and K^63^‐polyubiquitinated proteins were obtained using a pull‐down assay with beads conjugated with RAP‐80–UIM. Proteins pulled down with RAP‐80–UIM beads were analyzed by Western blotting with antibodies to K^63^‐linked ubiquitin. There was increased K^63^‐polyubiquitin chain formation within 5 minutes after injury (Figure 4A). A smeared pattern was observed, suggesting that a number of proteins may be ubiquitinated. The increase was also observed by immunohistochemistry with anti‐K^63^ ubiquitin antibody in porcine cartilage sections (Figure 4B). TAK‐1 was detected in the Rap‐80 UIM pull‐down assay (Supplementary Figure 1, available on the *Arthritis & Rheumatology* web site at http://onlinelibrary.wiley.com/doi/10.1002/art.39965/abstract).

### TAK‐1 from injured cartilage is linked to K^63^‐polyubiquitin but not to K^48^‐ or linear polyubiquitin as in TNF‐ or IL‐1–stimulated cartilage

**Figure 5:**
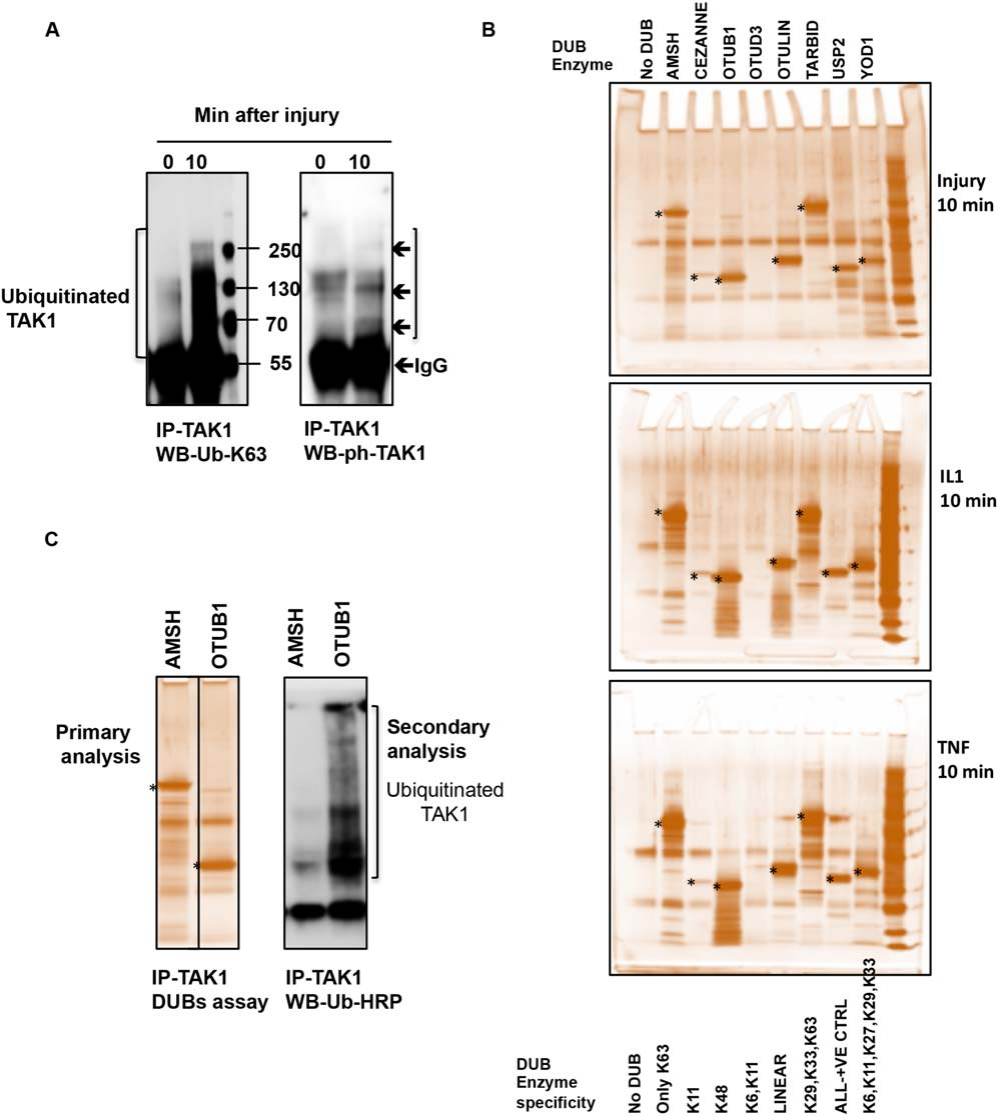
Analysis of polyubiquitin chains linked to transforming growth factor β–activated kinase 1 (TAK‐1) in injured cartilage compared to that stimulated with interleukin‐1 (IL‐1) or tumor necrosis factor (TNF). Porcine metacarpophalangeal joints were processed and cartilage lysates were prepared as described in Materials and Methods. **A,** TAK‐1 was immunoprecipitated (IP) and analyzed by Western blotting (WB) for K^63^‐linked ubiquitin (UB‐K^63^) or for TAK‐1 phosphorylated at T187. **B,** TAK‐1 was immunoprecipitated from injured cartilage, cartilage stimulated with IL‐1 for 10 minutes, and cartilage stimulated with TNF for 10 minutes. Immunocomplexes bound to beads were washed and then divided into 9 tubes and used in ubiquitin chain restriction enzyme analysis as described in Materials and Methods. A different deubiquitinase (DUB) was used in each tube as indicated. After incubation, supernatants were electrophoresed and gels were stained with silver to detect released ubiquitin chains (primary analysis). **Asterisks** indicate the position of recombinant deubiquitinase enzymes. +VE = positive. **C,** TAK‐1 was immunoprecipitated from injured cartilage and then treated with AMSH or otubain 1 (OUTB‐1) deubiquitinases as in **B** (primary analysis). Supernatants were analyzed by silver staining, and beads were mixed with 1× sample buffer and analyzed by Western blotting with anti‐ubiquitin antibody (secondary analysis). Representative results from 3 independent experiments are shown. Color figure can be viewed in the online issue, which is available at http://onlinelibrary.wiley.com/journal/doi/10.1002/art.39965/abstract.

Since there was an overall increase in K^63^‐polyubiquitination in response to injury, we investigated whether TAK‐1 is K^63^‐polyubiquitinated. TAK‐1 was immunoprecipitated from porcine cartilage lysates made at time point 0 or 10 minutes after dissection. Immunocomplexes were analyzed by Western blotting with anti‐K^63^ ubiquitin antibody and then reprobing with anti–phospho‐TAK‐1 antibody. Injury to porcine MCP joint cartilage caused K^63^‐polyubiquitination and phosphorylation of TAK‐1 in lysates made after injury (Figure 5A).

A UbiCREST kit was used to compare ubiquitin chain linkages attached to the TAK‐1 that was phosphorylated upon injury with those formed during IL‐1 and TNF stimulation. The kit uses a panel of linkage‐specific deubiquitinases to analyze the linkages present in polyubiquitin chains conjugated to a substrate protein 31. TAK‐1 was immunoprecipitated from porcine cartilage lysates made 10 minutes after injury or treatment with IL‐1 or TNF. Following deubiquitinase treatment, supernatants were analyzed by SDS‐PAGE and silver staining for the released ubiquitin (primary analysis), and the beads were Western blotted with anti‐ubiquitin antibody for conjugated ubiquitin (secondary analysis).

A strong polyubiquitin signal was observed in AMSH lanes (Figure 5B), indicating K^63^‐polyubiquitination of TAK‐1. However, no detectable signal was present in the OTUB‐1 lane, indicating that injury did not induce K^48^‐polyubiquitination of TAK‐1. Similarly, OTULIN produced no detectable mono‐ or polyubiquitin chains, demonstrating that TAK‐1 was not ubiquitinated by linear (Met1) linkages in response to injury. Polyubiquitin chains were also detected in the USP2‐positive control lane. Comparing this pattern of TAK‐1 polyubiquitination with those induced by IL‐1 and TNF (Figure 5B), we observed that the cytokines induced a strong polyubiquitin signal in AMSH and OTUB‐1 lanes, indicating that TAK‐1 is linked to K^63^‐ and K^48^‐polyubiquitin. OTULIN showed a positive signal of polyubiquitin chains only in the IL‐1 set, indicating that IL‐1 induced linear ubiquitination of TAK‐1 by a Met1‐E3 ligase. These results suggest differences in the polyubiquitin attached to TAK‐1 in response to injury, IL‐1 stimulation, and TNF stimulation. Secondary analysis of samples treated with AMSH and OTUB‐1 by anti‐ubiquitin Western blotting showed that the ubiquitin chains attached to TAK‐1 are mostly liberated by AMSH, while OTUB‐1 has no effect on the attached ubiquitin chains (Figure 5C), confirming that injury induced formation of K^63^‐ but not K^48^‐polyubiquitin chains on TAK‐1.

### Injury‐induced signaling is reduced upon avulsion of femoral epiphysis in cartilage‐specific TAK‐1–null mice but retained in TRAF‐6–null and MyD88‐null mice

**Figure 6:**
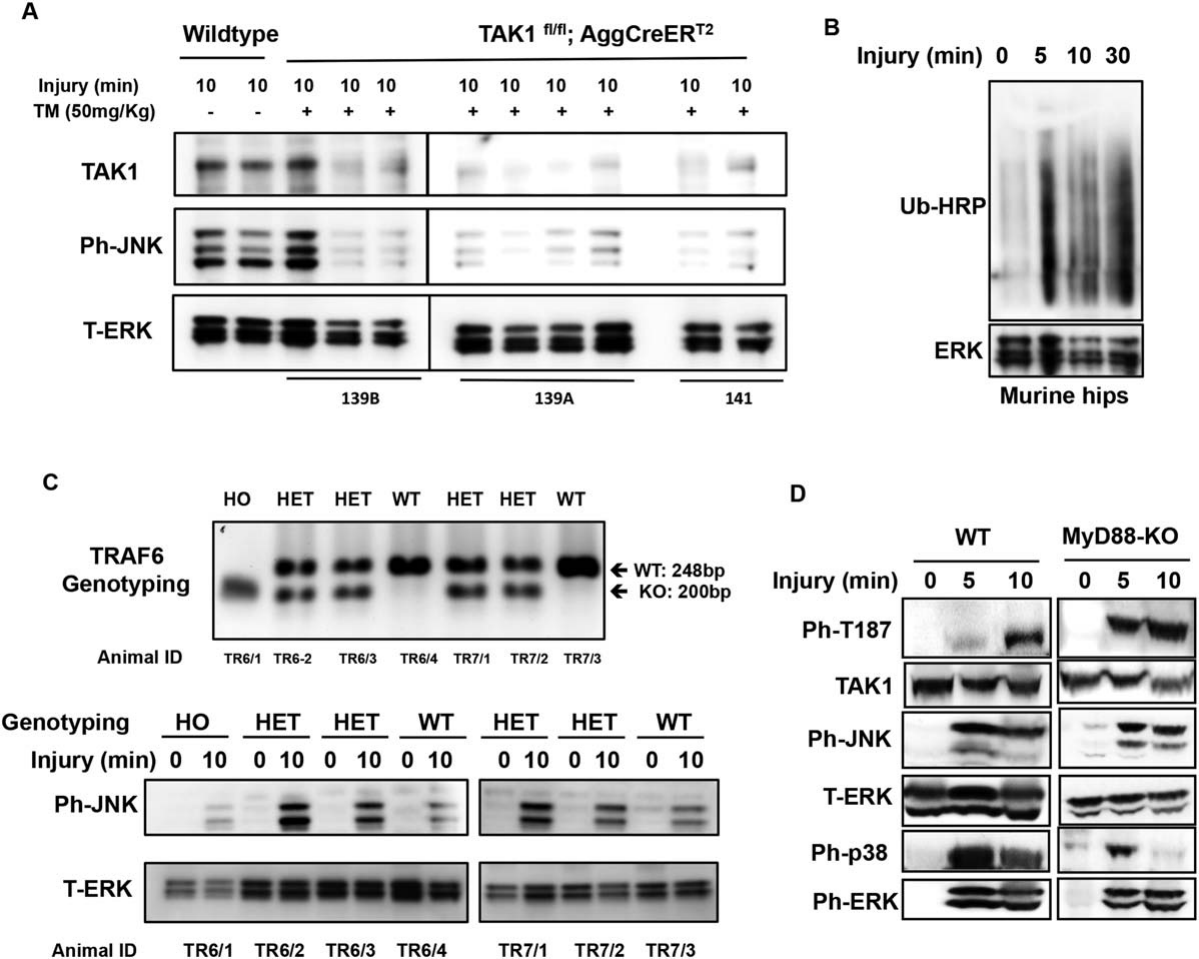
Injury‐induced signaling in avulsed murine epiphyses involves transforming growth factor β–activated kinase 1 (TAK‐1) and is retained in TRAF‐6–null and MyD88‐null mice. **A**, Cre recombination–mediated cartilage‐specific TAK‐1 deletion was induced by tamoxifen (TM; 50 mg/kg) as described in Materials and Methods. Murine proximal femoral epiphyses (2 hips per time point) were avulsed and either snap‐frozen (time point 0 minutes) or cultured for the indicated times in serum‐free medium. Cartilage lysates were analyzed by Western blotting for TAK‐1, phospho‐JNK (ph‐JNK), and total ERK (T‐ERK; loading control). **B**, Murine femoral epiphyses were avulsed, and lysates were analyzed by Western blotting for ubiquitinated proteins and ERK. Ub‐HRP = horseradish peroxidase–conjugated ubiquitin. **C**, TRAF‐6–null mice were generated as described in Materials and Methods. Top, DNA was extracted from the tips of the tails of 6‐day‐old animals for TRAF‐6 genotyping polymerase chain reaction. Bottom, Murine femoral epiphyses (1 hip per time point) were avulsed, and lysates were analyzed by Western blotting for phosphorylated JNK and total ERK (loading control). **D**, Murine femoral epiphyses from MyD88‐null mice were avulsed, and lysates were analyzed by Western blotting for phospho–TAK‐1, TAK‐1, phospho‐JNK, total ERK, phospho‐p38, and phospho‐ERK. HO = homozygous; HET = heterozygous; WT = wild‐type; KO = knockout.

The injury‐induced K^63^‐polyubiquitination of TAK‐1 in porcine cartilage indicates activation of an E2–E3 ligase complex. As previously described, TRAF‐6 and TRAF‐2 are the E3 ligases activated by IL‐1 and TNF, respectively 23, and TRAF‐6 also mediates signaling by a number of other stimuli. To explore further the upstream mechanisms of injury‐induced signaling, we investigated mice with avulsion injury of the proximal femoral epiphysis cartilage. This caused a pattern of TAK‐1 phosphorylation and laddering similar to that seen in porcine tissue (Figures 3A and B). To confirm the importance of TAK‐1, a mouse line expressing CreER under control of an aggrecan promoter was crossed with a TAK‐1 floxed mouse line. Treatment of the progeny with tamoxifen led to a cartilage‐specific deletion of TAK‐1 (constitutive knockout of TAK‐1 is fatal to the embryo). Avulsion of femoral epiphyses from these animals showed that TAK‐1 was needed for JNK activation (Figure 6A). This corroborated the results of the experiments with the inhibitor in porcine cartilage (Figure 2C). Western blotting of the mouse epiphyseal lysates showed that avulsion also caused polyubiquitination (Figure 6B).

To investigate whether TRAF‐6 is involved in injury‐dependent JNK activation, TRAF‐6–null mice were generated 29. Homozygous null animals lived a maximum of 9–10 days and represented <10% of the offspring. Avulsion experiments on TRAF‐6^−/−^, TRAF‐6^−/+^, and wild‐type animals were performed on day 6. JNK was activated by injury in all groups (Figure 6C), ruling out TRAF‐6 as the major injury‐induced E3 ligase.

MyD88 links signals from TLRs and IL‐1R1 to TRAF‐6 and downstream signaling. To investigate its possible involvement in injury‐dependent TAK‐1 activation, we tested MyD88‐null mice. The absence of MyD88 had no effect on TAK‐1 activation or MAPK signaling caused by avulsion (Figure 6D), indicating that Toll/IL‐1 receptors do not mediate the response, consistent with the findings of the TRAF‐6 experiments.

## DISCUSSION

The speed with which injury to cartilage activated signaling in chondrocytes in the present study was striking: IKK, IκB, ERK, and p38 MAP kinase were phosphorylated within 30 seconds. Phosphorylation of MKK‐4 was detectable by 1 minute, JNK by 2 minutes, and ATF‐2 by 5 minutes. Activation was propagated from the site of the cut made in the deep zone of the porcine MCP cartilage up into the mid‐zone, a distance of ∼20 chondrocyte lacunae (the tissue is ∼40 cells deep). Although we were unable to identify any soluble factor released from damaged cartilage to explain the effect, we cannot exclude the possible role of a highly unstable factor. The signal could be propagated between chondrocytes if there were connections between them. Chondrocytes appear solitary in their lacunae, but recent studies have shown long cytoplasmic extensions crossing the extracellular matrix, possibly enabling communication through gap junction channels formed by connexin 43 32, 33. However, it is still not understood how the cells sense the damage. The cell membrane is linked to the extracellular matrix, and it is possible that connective tissue cells directly detect changes in tension of the collagen fiber network.

To elucidate the upstream mechanism of this response, we investigated TAK‐1, because it mediates cell activation by several inflammatory stimuli. Injuring porcine or murine cartilage activated TAK‐1. TAK‐1 inhibition blocked NF‐κB and JNK activation and injury‐induced gene expression. In mice, knocking out TAK‐1 in cartilage prevented JNK activation in response to epiphyseal avulsion. Taken together, these findings showed that TAK‐1 plays an essential role in mediating the immediate response to injury.

Injury caused phosphorylation of TAK‐1 at T187 and S192 in both porcine and murine tissues, and it was present in high molecular weight forms, suggesting its ubiquitination. We found that K^63^‐polyubiquitination was occurring in the porcine cartilage upon injury and that TAK‐1 itself was K^63^‐polyubiquitinated.

We used deubiquitinases to analyze the ubiquitin chains on TAK‐1. Injury induced K^63^‐ but not K^48^‐ or linear polyubiquitination, while stimulation by IL‐1 or TNF caused both K^63^‐ and K^48^‐polyubiquitination. In addition, IL‐1 caused linear polyubiquitination. These distinct patterns suggest that the injury‐induced ubiquitin E3 ligase may be distinct from TRAF‐6 and TRAF‐2, which are induced by IL‐1 and TNF, respectively. Further analysis is required to detect the sites in TAK‐1 ubiquitinated by injury. K^63^‐polyubiquitination occurs on TAK‐1 at K158 and K562 in response to various stimuli including LPS, IL‐1, and TNF, and both sites are involved in the activation of the kinase 22, 23, 34, 35, 36, 37. However, TAK‐1 can be activated by unanchored and linear polyubiquitin 38, 39.

TAK‐1 is activated by other injuries in other tissues. It was phosphorylated in satellite cells upon skeletal muscle injury induced by barium chloride and promoted myofiber regeneration 40. TAK‐1 was also activated in brain injury following experimental subarachnoid hemorrhage or trauma, and its inhibition reduced downstream signaling and neurologic deficits 41, 42.

Since TRAF‐6 is the E3 ligase mediating a number of stimuli, we investigated TRAF‐6–knockout mice. Few viable homozygous null offspring were obtained, but their femoral epiphyses showed JNK activation upon avulsion. Epiphyses from MyD88‐knockout animals also responded to injury, indicating that TLRs and IL‐1R have no major role in the response. These results, taken together with the differences in TAK‐1 ubiquitination caused by cytokines and injury, suggest involvement of an E3 ligase other than TRAF‐6. Besides TRAF‐2, E3 ligases known to generate K^63^‐polyubiquitin include tripartite motif–containing protein 25 (TRIM‐25) 43, TRIM‐8 44, and mind bomb 2 45. The E2‐conjugating enzyme is also unknown. Research on E2 and E3 enzymes involved in TAK‐1 activation is limited, and proteomics analyses are needed to detect the enzymes involved in the injury response.

## AUTHOR CONTRIBUTIONS

All authors were involved in drafting the article or revising it critically for important intellectual content, and all authors approved the final version to be published. Dr. Ismail had full access to all of the data in the study and takes responsibility for the integrity of the data and the accuracy of the data analysis.

### Study conception and design

Ismail, Saklatvala.

### Acquisition of data

Ismail, Didangelos.

### Analysis and interpretation of data

Ismail, Vincent, Saklatvala.

## Supporting information


**Supplementary Figure 1: TAK1 is identified in Rap80‐ UIM pull down in response to cartilage injury.** Cartilage was dissected and either snap frozen (0 min) or cultured for the indicated time points. K63‐ubiquitin linked proteins were pulled down from cartilage lysates by Rap80 UIM as described in Methods and proteins bound to the beads were western blotted with an anti‐ K‐63 ubiquitin antibody and anti‐TAK1 antibody. Lysates were western blotted for ph‐JNK and ERK.
