# Supplementary material for: Rapid Activation of Transforming Growth Factor β–Activated Kinase 1 in Chondrocytes by Phosphorylation and K63‐Linked Polyubiquitination Upon Injury to Animal Articular Cartilage
Source: Arthritis Rheumatol. 2017 Feb 27;69(3):565–75. doi: 10.1002/art.39965 (PMC5347887; doi:10.1002/art.39965)
Supplement: Supplementary file 1 — Supplementary Figure 1: TAK1 is identified in Rap80‐ UIM pull down in response to cartilage injury. Cartilage was dissected and either snap frozen (0 min) or cultured for the indicated time points. K63‐ubiquitin linked proteins were pulled down from cartilage lysates by Rap80 UIM as described in Methods and proteins bound to the beads were western blotted with an anti‐ K‐63 ubiquitin antibody and anti‐TAK1 antibody. Lysates were western blotted for ph‐JNK and ERK. [file ART-69-565-s001.pdf]

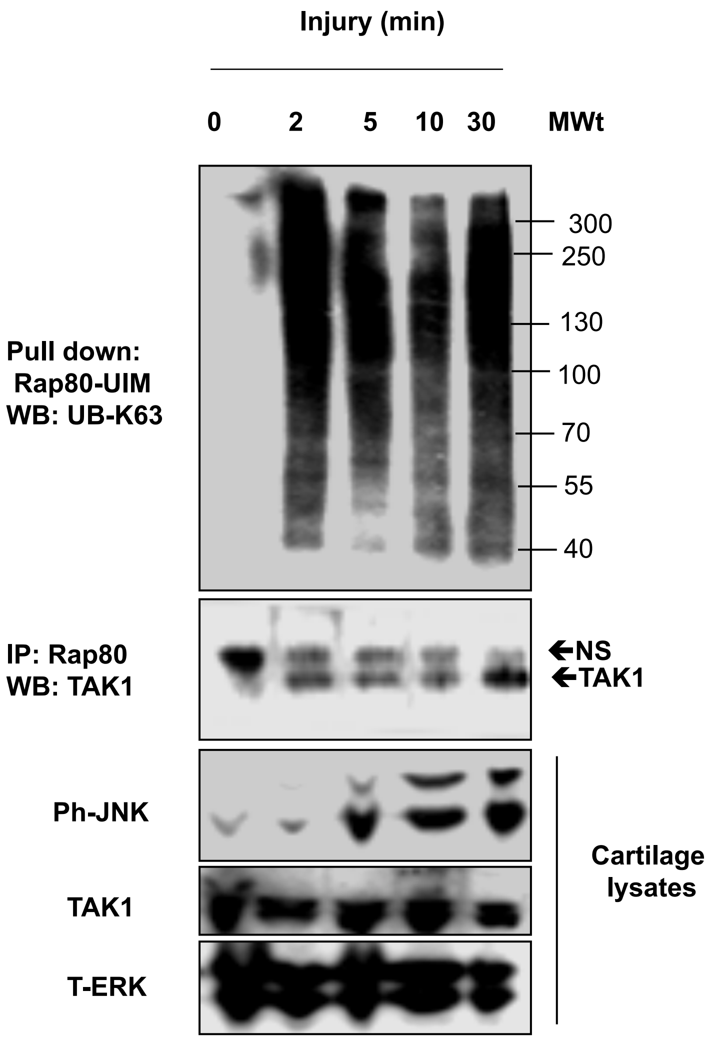

Supplementary Figure 1: TAK1 is identified in Rap80- UIM pull down in response to cartilage injury. Cartilage was dissected and either snap frozen (0 min) or cultured for the indicated time points. K63-ubiquitin linked proteins were pulled down from cartilage lysates by Rap80 UIM as described in Methods and proteins bound to the beads were western blotted with an anti- K-63 ubiquitin antibody and anti-TAK1 antibody. Lysates were western blotted for ph-JNK and ERK.
